# Benefits of diffusion-weighted imaging in pediatric acute osteoarticular infections

**DOI:** 10.1007/s00247-022-05329-3

**Published:** 2022-04-04

**Authors:** Céline Habre, Paul Botti, Méryle Laurent, Dimitri Ceroni, Seema Toso, Sylviane Hanquinet

**Affiliations:** 1 Pediatric Radiology Unit, Radiology Division, Diagnostic Department, Children’s Hospital, University Hospitals of Geneva, CH-1211 Geneva 14, Switzerland; 2 Pediatric Orthopedics Unit, Surgery Division, Department of Women-Children-Teenagers, Children’s Hospital, University Hospitals of Geneva, Geneva, Switzerland

**Keywords:** Abscess, Children, Diffusion-weighted imaging, Magnetic resonance imaging, Osteoarticular infection, Osteomyelitis, Septic arthritis

## Abstract

**Background:**

Contrast-enhanced magnetic resonance imaging (MRI) is recommended for the diagnosis of acute osteoarticular infections in children. Diffusion-weighted imaging (DWI) may be an alternative to the injection of gadolinium.

**Objective:**

To evaluate unenhanced MRI with DWI in comparison to contrast-enhanced MRI for the diagnostic work-up of acute osteoarticular infections in children.

**Materials and methods:**

This retrospective study included 36 children (age range: 7 months-12 years) with extra-spinal osteoarticular infections and MRI performed within 24 h of admission. MRI protocol included short tau inversion recovery (STIR), water-only T2 Dixon, T1, DWI, and gadolinium-enhanced T1 sequences. Two readers reviewed three sets of images: 1) unenhanced sequences, 2) unenhanced sequences with DWI and 3) unenhanced followed by contrast-enhanced sequences (reference standard). Sensitivity and specificity of sets 1 and 2 were compared to set 3 and assessed to identify osteoarticular infections: osteomyelitis (long bones, metaphyseal equivalents), septic arthritis and abscess (soft tissues, bone).

**Results:**

All 14 cases of osteomyelitis in the metaphyses and diaphyses of long bones and all 27 cases of septic arthritis were identified by unenhanced sequences, but 4/16 abscesses were missed. For the diagnosis of abscess, DWI increased sensitivity to 100%. Among the 18 osteomyelitis in metaphyseal equivalents, 4 femoral head chondroepiphyses were identified by contrast-enhanced sequences only.

**Conclusion:**

MRI for suspected pediatric acute osteoarticular infections is the best diagnostic modality to guide patient management. An unenhanced protocol with DWI may be an alternative to a contrast-based protocol, even in the presence of an abscess. However, gadolinium remains necessary to assess for chondroepiphyseal involvement of the femoral head.

**Supplementary Information:**

The online version contains supplementary material available at 10.1007/s00247-022-05329-3.

## Introduction

Acute pediatric osteoarticular infections bear significant morbidity if diagnosis and treatment are delayed, with potentially irreversible damage to the growing skeleton [1–3]. The need for prompt imaging and surgery is well documented and magnetic resonance imaging (MRI) holds a central role in current clinical algorithms [4, 5]. When acute osteoarticular infection is suspected in a child, emergency MRI aims to identify bone marrow signal abnormality, joint effusion and collections that can be targeted by puncture and aspiration to achieve a definite diagnosis [6, 7]. Alternatively, imaging findings will suggest a differential diagnosis and redirect management [8].

Contrast-enhanced MRI is the standard of imaging in children with suspected acute osteoarticular infection [9, 10]. However, safety concerns regarding the adverse effects of gadolinium retention in children raise the question of gadolinium-based contrast injection in pediatric MRI [11–14] . An unenhanced protocol that could confirm musculoskeletal infection and assist in the decision for surgical management is desirable.

Diffusion-weighted imaging (DWI) relates to tissue and fluid composition, such as high cellularity and viscosity of pus [15]. It has proven helpful in musculoskeletal imaging of adults for the diagnosis of soft-tissue abscess [16], osteomyelitis of the diabetic foot [17] and spine infections [18]. As for pediatric musculoskeletal imaging, previous studies have investigated the role of DWI in juvenile idiopathic arthritis [19, 20], chronic recurrent multifocal osteomyelitis [21] and bone tumors [22–24] but not for acute ostearticular infections.

The objective of our study was to assess whether DWI can replace contrast injection for the diagnosis of osteomyelitis, septic arthritis and abscess in children.

## Materials and methods

This retrospective study was conducted in a single pediatric radiology center from January 2015 to March 2020 and was approved by our hospital’s local ethics committee (CE 14-102R).

We reviewed all consecutive MRIs performed before treatment of children admitted for suspected acute peripheral osteoarticular infection. Children with known hematological or oncological disease were excluded. Electronic medical records were searched for clinical and biological data.

MRI were performed on a 1.5-T unit (Avanto; Siemens, Erlangen, Germany) using the following protocol: T1-weighted (T1-W) turbo spin-echo (one longitudinal plane); two orthogonal planes with T2-weighting plus fat suppression (T2-W FS), short tau inversion recovery (STIR) (longitudinal plane) and water-only data set of fast spin-echo T2-weighted Dixon sequences (axial plane); DWI (axial plane) using single shot echo planar imaging (EPI), acquired using two *b* values, 0 and 800 s/mm^2^, with automatic monoexponential calculation of apparent diffusion coefficient (ADC) maps; and post-contrast injection T1-weighted spin echo with frequency-selective fat saturation (two orthogonal planes). Post-contrast sequences were obtained after injection of 0.2 ml/kg of gadoteric acid (Dotarem, Guerbet, France).

In young children, it may be difficult to localize the origin of infection because of ill-defined and nonspecific clinical symptoms. Therefore, MRI consistently started with a 3-D extended field of view (FOV) STIR to identify the anatomical site of signal abnormality. Subsequent sequences were acquired with a smaller FOV and with a dedicated peripheral coil targeted at the region of interest.

The approximate duration of MRI was 30 min, of which 15 to 20 min were dedicated to T1-W and T2-W FS sequences, 2 to 4 min were dedicated to DWI acquisition and 15 min to contrast injection, i.e. monitoring of venous catheter, manual injection and acquisition in two planes (acquisition time range: 7–11 min). General anesthesia was performed for children younger than 6 years old. Online Supplementary Material 1 includes a table with acquisition parameters depending upon the FOV dimension chosen to fit the anatomical location.

### MRI analysis

All MRIs were anonymised, coded, transferred to and stored in random order on a dedicated computer station with OsiriX MD v 12.0.1 software (Geneva, Switzerland).

Image analysis was performed independently by two pediatric senior radiologists (C.H. and S.T., with 3 and 7 years of paediatric MRI experience, respectively), blinded to the clinical features, radiologic reports and final diagnoses, after a consensus-based training period on 10 patients. Disagreements were resolved by consensus.

Three sets of MRI sequences were compared:

1. Unenhanced sequences (STIR, T2-W FS, T1-W): either STIR or water-only images of T2-W Dixon could be selected for analysis.
2. Unenhanced sequences + DWI sequences.
3. Unenhanced sequences + post-contrast sequences (T1-W FS).

The third combination was defined as the reference standard in keeping with relevant literature [9]. Each set of images was read successively without interval and was given a score immediately reported in a table.

Osteoarticular infections were divided into four patterns:

1. Osteomyelitis in long bones (metaphyses and diaphyses).
2. Osteomyelitis in metaphyseal equivalents (i.e. carpal and tarsal bones, margins of epiphyseal and apophyseal centers, and nonepiphyseal end of short tubular bones of the hands and feet). Although small round bones are epiphyseal equivalents, their ossifying centers behave like metaphyseal equivalents in the vicinity of the acrophysis along which enchondral growth occurs, hence for reasons of simplication, we defined them as metaphyseal equivalents [9, 25, 26]
3. Septic arthritis.
4. Abscess in bone or soft tissue (subperiosteal compartment, muscle and subcutaneous compartment).

The definition of infection on MRI was established on the basis of the existing literature [9, 14–16]. Accordingly, abnormal signal of bone, joint and cartilage (growth cartilage and epiphyses) was regarded as hypointense on T1-W sequences and hyperintense on fluid-sensitive sequences. Enhancement could be either increased (bone marrow in osteomyelitis, synovium in arthritis) or decreased (non-enhancing center with rim enhancement in abscess, enhancement defects in growth and epiphyseal cartilage). On DWI, osteomyelitis was characterized by high signal at b=800 and high signal on the apparent diffusion coefficient (ADC) map; joint effusion was characterized by low signal at b=800 and high signal on the ADC map, with opposite signal in case of purulent content; abscess was defined as a well-defined area of restricted diffusion, with high signal at b=800 and low signal on the ADC map. DWI was only assessed on a visual basis with correlation between the images acquired at high b value and the corresponding ADC map, without ADC quantification. When possible, comparison with the opposite side or limb was used to decide normal or abnormal signal.

A binary score was used for each MRI sequence to assess the absence (score 0) or the presence (score 1) of abnormal signal.

### Statistical analysis

Descriptive analysis was used for patient characteristics and diagnosed pathologies. Sensitivity and specificity with 95% confidence interval (CI) were calculated to detect each of four patterns of osteoarticular infection on images in sets 1 and 2. All statistical analyses were performed using MedCalc for Windows (MedCalc Software, Ostend, Belgium). Inter-rater agreement was calculated using Kappa statistics, with level of agreement defined as almost perfect for values >0.90, strong 0.80–0.90 and moderate 0.60–0.79 [27].

## Results

### Patient characteristics

Our cohort included 38 children with proven osteoarticular infections for whom MRI was performed on admission. After excluding 2 cases because of image distortion on DWI, 36 patients were included (16 F/20 M), with a median age of 26 months (range: 7 months-12 years). Twenty-six children were younger than 5 years old. Symptoms lasted from 1 to 15 days (median: 3 days) before admission. Patient characteristics and clinical data are summarised in Table 1.

**Table 1:** Patient characteristics and clinical data

	*n*=36
Age (months), mean±SD	45 ± 43
Gender (F/M)	16/20
T > 38 °C (n)	14 (39%)
WBC count (G/L), mean±SD	12,7 ± 4.3
Platelet count (G/L), mean±SD	377 ± 143
CRP (mg/l), mean±SD	41,8 ± 41
Duration of symptoms (days), mean±SD	3,8 ± 2.9

*CRP* C-reactive protein, *F* female, *G/L* Gigaliter, *mg/l* milligrams/liter, *M* male, *n* number of patients, *SD* standard deviation, *T* temperature, *WBC* white blood cell

All MRIs were performed within 24 h of admission (range: 30 min–22 h 20 min, median: 3 h 5 min). Twenty-six patients (72%) underwent a surgical procedure following imaging, either for microbial identification or drainage/debridement. Pathogenic bacteria were identified in 32 patients, either by culture from bone, joint or blood samples, or by polymerase chain reaction assay on oropharyngeal swab: *Kingella Kingae* (56%), *Methicillin-susceptible Staphylococcus aureus* (22%), *Streptococcus* sp.*(8%)*, *Staphylococcus epidermidis (3%)*. In the remaining four children, the diagnosis of osteoarticular infection was made on the basis of clinical and biological findings and favorable evolution under empiric antibiotic therapy.

Of the 32 cases of osteomyelitis in long bones and metaphyseal equivalents, 27 cases of septic arthritis and 16 bone/periosteal abscesses were identified. The anatomical regions involved were, in decreasing order, the hip (9), the ankle (7), the foot (7), the knee (6), the elbow (3), the pelvis (2), the hand (1) and the shoulder (1). Each patient was scanned once but may have displayed more than one pattern of infection. Table 2 shows sensitivity and specificity of unenhanced and DWI sequences depending on the pattern of infection.

**Table 2:** Sensitivity and specificity of conventional unenhanced sequences alone, and of unenhanced sequences with diffusion-weighted imaging, in comparison to post-contrast sequences. Percentages in brackets indicate 95% confidence intervals. Long bones include metaphyseal and diaphyseal involvement. Metaphyseal equivalents designate carpal and tarsal bones, epiphyseal and apophyseal centers, and non-epiphyseal end of short tubular bones. Abscesses are divided into soft tissue and bone locations

	**Sensitivity**				
	Osteomyelitis		Arthritis	Abscess	
	Long bones (*n*=14)	Metaphyseal equivalents (*n*=18)	(*n*=27)	Soft tissue (*n*=10)	Bone marrow (*n*=6)
Unenhanced sequences	100.0%	77.8%	100.0%	80.0%	66.7%
(T1, STIR/T2 with FS)	(76.8–100.0%)	(52.4–93.6%)	(87.2–100.0%)	(44.4%–97.5%)	(22.3–95.7%)
Unenhanced sequences + DWI	100.0%	77.8%	100.0%	100.0%	100.0%
	(76.8–100.0%)	(52.4–93.6%)	(87.2–100.0%)	(69.2–100.0%)	(54.1–100.0%)
	**Specificity**				
	Osteomyelitis		Arthritis	Abscess	
	Long bones (*n*=22)	Metaphyseal equivalents (*n*=18)	(*n*=9)	Soft tissue (*n*=26)	Bone marrow (*n*=30)
Unenhanced sequences	100.0%	100.0%	100.0%	96.2%	96.7%
(T1, STIR/T2 with FS)	(84.6–100.0%)	(81.5–100.0%)	(66.4–100.0%)	(80.4–99.9%)	(82.8–99.9%)
Unenhanced sequences + DWI	100.0%	100.0%	100.0%	96.2%	90.0%
	(84.6–100.0%)	(81.5–100.0%)	(66.4–100.0%)	(80.4–99.9%)	(73.5–97.9%)

*DWI* diffusion-weighted imaging, *FS* fat suppression, *STIR* short tau inversion recovery

### Diagnostic MRI

#### Osteomyelitis in long bones

Fourteen patients (39%) had osteomyelitis in long bone metaphyses or diaphyses, distributed as follows: 6 femurs, 3 fibulas, 2 tibias, 2 ulnas and 1 metatarsal bone. All cases were identified on standard unenhanced sequences with no added value of DWI.

#### Osteomyelitis in metaphyseal equivalents

**Fig. 1:**
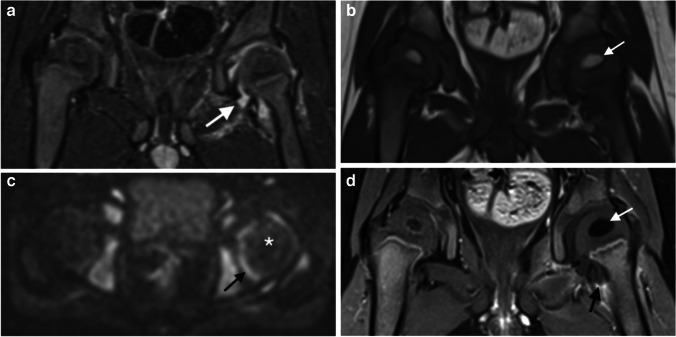
A 10-month-old boy with left femoral chondroepiphyseal injury from left hip arthritis. **a** Coronal STIR demonstrates joint effusion alone (*arrow*). **b** On the coronal T1-weighted image, the ossification center of the femoral head is unremarkable (*arrow*). **c** Axial DWI (b value of 800 s/mm^2^) confirms joint effusion (*arrow*), but the femoral head has normal signal in comparison to the right side (*asterisk*). **d** Coronal T1-weighted fat-suppressed image after contrast injection demonstrates absent enhancement of left femoral chondroepiphysis (*white arrow*); there is enhancement around the joint capsule consistent with arthritis (*black arrow*). DWI diffusion-weighted imaging, STIR short tau inversion recovery

Eighteen patients (50%) had osteomyelitis in a metaphyseal equivalent, distributed as follows: 11 epiphyses, 3 tarsal bones, 2 carpal bones, 1 pubic bone close to the triradiate cartilage and 1 iliac bone close to the sacroiliac joint. The 11 affected epiphyses (median age: 20 months) involved the ossification centers of 5 femoral heads, 3 distal tibias, 2 distal femurs and 1 proximal humerus. Of the 18 metaphyseal equivalent osteomyelites, 14 were identified on standard unenhanced sequences with no additive value of DWI. All 4 missed locations were the femoral head chondro-epiphysis, in patients ages 9, 10, 13 and 30 months old. Abnormal signal indicative of injury was only depicted as absent enhancement on contrast-enhanced sequences (Fig. 1).

#### Septic arthritis

Among the 27 patients with arthritis, 7 (26%) were isolated and 20 (74%) were concurrent with osteomyelitis. All cases were identified on standard unenhanced sequences with no added value of DWI.

#### Abscesses

##### Soft-tissue abscesses

**Fig. 2:**
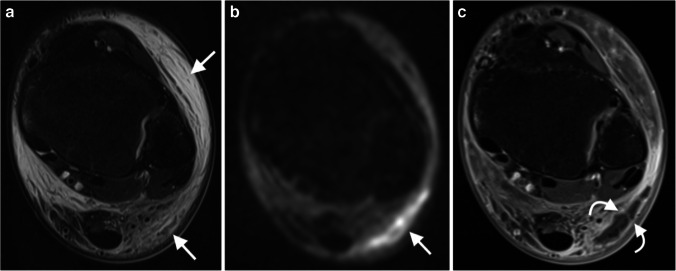
A 12-year-old girl with a soft-tissue abscess associated with arthritis and cellulitis of the ankle. **a** Axial STIR shows ill-defined and circumferential oedema of the soft tissues around the ankle (*arrows*). **b** Axial DWI (b value of 800 s/mm^2^) allows delineation of a circumscribed subcutaneous collection (*arrow*). **c** An axial T1-weighted fat-suppressed image after contrast injection confirms a subcutaneous abscess (*curved arrows*). DWI diffusion-weighted imaging, STIR short tau inversion recovery

Ten patients (28%) had an abscess in a soft-tissue location (i.e. in subcutaneous soft tissue, subperiosteal space and tendon sheath). Of these, eight were identified on standard unenhanced sequences. DWI identified all cases (Fig. 2).

##### Bone abscesses

**Fig. 3:**
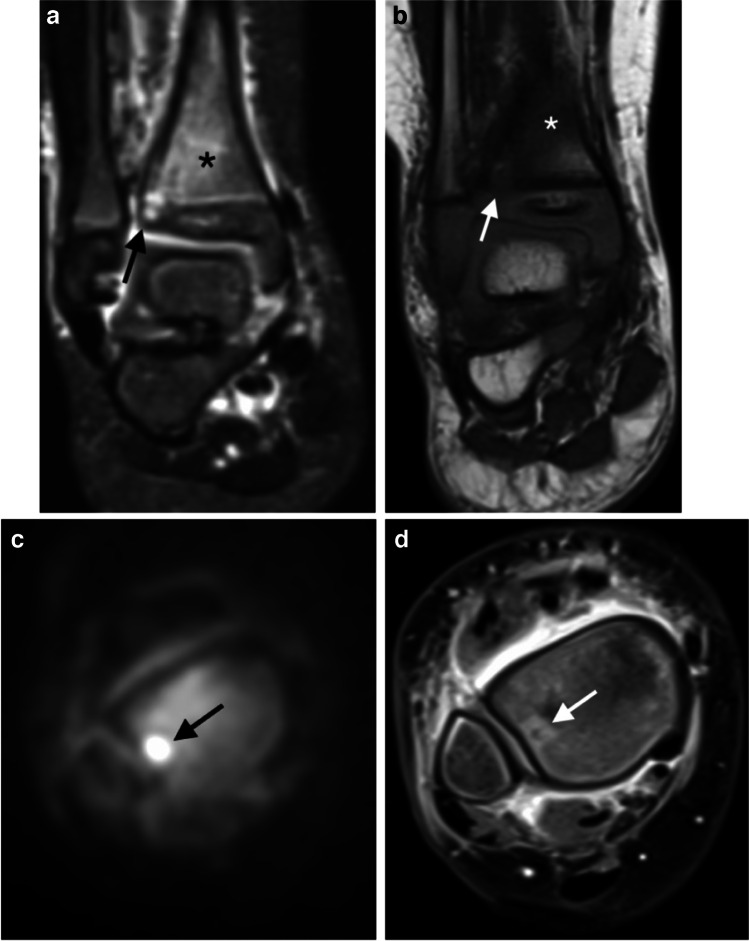
A 20-month-old boy with bone abscess in osteomyelitis of the distal tibia. **a** Coronal STIR shows a focus of high signal of the metaphysis and physis of the distal tibia (*arrow*), within a background of medullary hyperintensity, consistent with osteomyelitis (*star*). **b** A coronal T1-weighted image shows corresponding focal hypointense signal (*arrow*) and diffuse hypointense metaphysis (*star*). **c** Axial DWI (b value of 800 s/mm^2^) depicts a focus of hyperintensity (*arrow*). **d** An axial T1-weighted fat-suppressed image after contrast injection confirms a small bone abscess with peripheral enhancement around a low signal core (*arrow*). DWI diffusion-weighted imaging, STIR short tau inversion recovery

Six patients (17%) had an abscess in the bone medullary cavity. Of these, four were identified on standard unenhanced sequences. In all six cases, DWI was positive for the diagnosis of an abscess (Fig. 3).

#### Inter-reader agreement

Global inter-reader agreement was strong with a Kappa coefficient of 0.826 (95% CI, 0.78–0.88). Kappa coefficients ranged from 0.68 to 0.92 for the conventional unenhanced sequences, from 0.72 to 0.87 for DWI and from 0.71 to 0.87 for post-contrast sequences, depending on the pattern of infection.

## Discussion

Magnetic resonance imaging is the modality of choice for investigating acute osteoarticular infections in children, decision-making about antibiotic therapy and surgical management [4–6].

Previous pediatric studies showed that T1-W and fluid-sensitive (STIR and T2-W with fat suppression) sequences are sufficient to confirm or exclude osteomyelitis and arthritis, but gadolinium injection remains the reference standard to identify complications of osteomyelitis such as abcess, and growth cartilage and chondroepiphyseal injury [9, 10, 13, 14, 28]. Averill et al. [13] retrospectively studied the MRIs of 78 children with suspected osteoarticular infection and demonstrated equal sensitivity and specificity between pre-contrast and post-contrast images for the diagnosis of osteomyelitis. However, reader confidence for abscess was increased with contrast injection. Similarly, a more recent analysis of 90 pediatric MRIs by Markhardt et al. [14] showed that fluid-sensitive sequences were as sensitive as contrast-enhanced sequences with a 100% negative predictive value for osteoarticular infection, although the diagnosis of concomitant abscess was missed in three cases without contrast injection. Other authors reviewed MRI studies of 14 epiphyseal osteomyelitis cases in children in correlation with surgery [28]. Among the 10/14 abnormally enhanced epiphyses, 4 corresponded to either chondroepiphyseal abscess or injury without abscess, highlighting that an enhancement defect may indicate chondroepiphyseal involvement. In line with these studies, current MRI guidelines recommend witholding gadolinium injection when pre-contrast sequences are normal, and reserving contrast medium for suspected abscess and/or epiphyseal involvement [9, 10].

To avoid gadolinium injection, we evaluated the diagnostic performance of an unenhanced protocol with DWI in comparison with a standard contrast-enhanced MRI. To our knowledge, DWI is not currently part of routine pediatric MRI protocols for osteoarticular infection in most centers, although recently, Chaturvedi [29] reported the usefulness of DWI to assess for subperiosteal abscess in children based on unpublished data. So far, only adult studies have emphasized that ADC values can differentiate osteomyelitis from noninfectious bone marrow edema [17, 30, 31].

In our study, all cases of osteomyelitis and arthritis were correctly identified by unenhanced sequences. The addition of DWI increased the sensitivity of unenhanced sequences for the detection of abscess, by demonstrating bright signal on high *b* value images with corresponding low intensity on ADC map. Interestingly, we report one 11-year old boy with ankle osteomyelitis complicated by bone necrosis for which DWI showed restricted diffusion, but contrast-enhanced sequences did not confirm bone abscess. We considered this case a false-positive bone abscess on DWI. Importantly, in the absence of contrast medium, this misdiagnosis would not have altered the patient’s management. In either case, bone abscess and bone necrosis, surgical debridement is necessary to improve antibiotic therapy efficiency [13].

Another important issue shown by our study is that DWI is less sensitive in detecting femoral head chondroepiphysial involvement in children younger than the age of 30 months. This age group has an increased propensity for chondroepiphyseal infection [9, 26, 30]. The femoral head frequently does not enhance after contrast injection in hip infection. The reason for this peculiar behavior on MRI remains controversial and may be related to direct infection or to ischemic phenomena concomitant with joint infection [32–34]. In our cohort, four diseased femoral heads were missed on non-enhanced and DWI images. There is an age-related increase in fat marrow content and parallel decrease of enhancement of the epiphyses [35]*.* This may partly explain why non-contrast images are less sensitive to abnormal epiphyseal signal in the younger patient, whose epiphyses contain less fat. Moreover, the small size of growing epiphyseal ossification centers may negatively affect spatial resolution and render difficult the detection of any abnormal signal. Similarly, we presumed that DWI could not identify altered signal of the femoral head chondroepiphyses because of their fatty, marrow-rich and low proton density content, which makes them appear normally hypointense on DWI and ADC map [15, 36]. In our series, the other seven epiphyses showed abnormal signal on pre-contrast sequences and therefore contrast-enhanced sequences had no added value for the diagnosis of chondritis and osteomyelitis.

Our study is limited by its retrospective nature and its small sample size. However, it is notable for the very young age of our cohort (72% were younger than 5 years old) for whom MRI, without contrast injection, could be of increased interest. One reason for the limited number of patients is that we already regularly dispense with contrast medium in MRI studies when pre-contrast and DWI sequences are informative enough to enable the decision for surgical management and to guide the procedure. The retrospective nature of our work, along with the absence of a control group, could have been the source of bias by positively influencing the interpretation in cases of uncertainty. Regarding DWI, we only analysed images on a qualitative basis, as either increased or decreased signal with visual correlation to the ADC map, without measuring ADC values. Although our inter-reader agreement was high, the visual analysis of DWI may lead to subjective interpretation in ill-trained radiologists. It may be even more difficult when small bones of the extremities are examined, where cumulative dephasing from increased K space filling to increase resolution can be responsible for image distorsion. In our study, we chose the axial plane for DWI to minimize geometric distortion.

Another limitation of our study relates to its reproducibility in other institutions. In our pediatric hospital, we are able to perform an emergent MRI any time septic arthritis or osteomyelitis is suspected, but we are aware that MRI and general anesthesia are not always available during night shifts and out-of-hours in every hospital. Finally, we emphasize that our study was designed to test DWI as part of an MRI protocol for invstigating osteoarticular infection, and not to differentiate infection from alternative diagnoses, such as Langerhans cell histiocytosis.

Our study acts as a reminder that all MRI protocols should be tailored to each individual patient. The decision to inject contrast medium should be undertaken while the unenhanced images are acquired. To that end, we encourage the routine addition of DWI to standard unenhanced sequences for the work-up of osteoarticular infections in children and suggest the following recommendations:

- As currently and widely accepted, when T1- and T2-W FS are negative, there is no need for DWI and contrast medium.
- When T1- and T2-W FS are positive, DWI should be performed; if DWI does not show restricted diffusion, then contrast medium is not required.
- However, if DWI shows restricted diffusion in soft tissue or bone, then the likelihood of an abscess is high. Depending on the radiologist’s level of confidence, contrast medium should be considered to increase the accuracy of findings. Alternatively, the radiologist may chose to abstain from injecting contrast medium when images are informative enough to guide the orthopedist’s surgical procedure.

In addition, contrast-enhanced sequences should be maintained for suspected proximal femoral head involvement especially in young children, irrespective of the suspected underlying mechanism (direct infection, transient ischemia) since it impacts decision-making regarding joint aspiration, duration of antibiotic therapy and clinical follow-up. It would be useful to perform multicenter studies in a larger cohort to obtain data that would support our recommandations and our belief that contrast can be avoided in many children with this common acute pathology.

## Conclusion

Emergency unenhanced MRI with DWI can establish the diagnosis of acute osteoarticular infections in children and negates the need for gadolinium. Such a protocol is sufficient for prompt medical and surgical management. However, gadolinium-based contrast injection remains necessary to evaluate the femoral head chondroepiphysis in younger children, in whom diffusion imaging is not reliable.

## Supplementary Information

ESM 1 **Summary of acquisition parameters depending on the size of field-of-view and coil, chosen to fit the patient and the location of disease.** In children younger the 6 years old, the initial sequence is an extended field-of-view three-dimensional short tau inversion recovery sequence. (DOCX 21.5 kb)
