# Supplementary material for: Benefits of diffusion-weighted imaging in pediatric acute osteoarticular infections
Source: Pediatr Radiol. 2022 Apr 4;52(6):1086–94. doi: 10.1007/s00247-022-05329-3 (PMC9107444; doi:10.1007/s00247-022-05329-3)
Supplement: Supplementary file 1 — Summary of acquisition parameters depending on the size of field-of-view and coil, chosen to fit the patient and the location of disease. In children younger the 6 years old, the initial sequence is an extended field-of-view three-dimensional short tau inversion recovery sequence. (DOCX 21.5 kb) [file 247_2022_5329_MOESM1_ESM.docx]

|  | **Plane** | | | **Field of view** | **Matrix** | | **In plane resolution (mm)** | **Slice thickness (mm)** | **Gap** | **Echo time (ms)** | **Repetition time**  **(ms)** | **GRAPPA**  **factor** | **Bandwidth (Hz/Px)** | **Acquisition**  **time**  **(min:s)** |
| --- | --- | --- | --- | --- | --- | --- | --- | --- | --- | --- | --- | --- | --- | --- |
| **T2 STIR SPACE** | | 3D | 200 x 200 | | 256 x 256 | | 0.8 x 0.8 x 0.8 | 0.8 | - | 155 | 2000 | 2 | 444 | 04:02 |
|  | | |  | | | | | | | | | | | |
| **Large FOV (body coil 18 CH)** | | |  | | | | | | | | | | | |
| T2 STIR | | coronal | 200 x 200 | | 320 x 240 | | 0.6 x 0.6 x 4.0 | 4 | 0.8 | 56 | 4050 | 2 | 289 | 04:09 |
| T1 TSE | | coronal | 200 x 138 | | 256 x 192 | | 0.8 x 0.8 x 4.0 | 4 | 0.4 | 9.9 | 524 | 2 | 250 | 02:07 |
| T2 DIXON | | axial | 200 x 231 | | 384 x 384 | | 0.5 x 0.5 x 3.0 | 3 | 0.3 | 58 | 6360 | 2 | 200 | 06:42 |
| EPI diffusion b0_800 | | coronal | 300 x 340 | | 148 x 148 | | 2.0 x 2.0 x 4.0 | 4 | 0.4 | 69 | 3500 | - | 2252 | 02:38 |
| After contrast injection : | |  |  | |  | |  |  |  |  |  |  |  |  |
| T1 SE fat saturation | | coronal | 200 x 200 | | 256 x 256 | | 0.4 x 0.4 x 3.0 | 3 | 0.3 | 11 | 468 | 2 | 183 | 04:03 |
| T1 SE fat saturation | | axial | 200 x 200 | | 320 x 320 | | 0.6 x 0.6 x 3.0 | 3 | 0.3 | 11 | 490 | 2 | 182 | 03:10 |
| **Medium FOV (flex or ankle coils)** | | | | | |  | | | | | | | | |
| T2 STIR | | sagittal | 150 x 150 | | 256 x 256 | | 0.6 x 0.6 x 0.8 | 0.8 | - | 167 | 2000 | 2 | 444 | 06:30 |
| T1 TSE | | coronal | 170 x 170 | | 384 x 384 | | 0.4 x 0.4 x 3.0 | 3 | 0.3 | 12 | 526 | - | 151 | 03:13 |
| T2 DIXON | | axial | 130 x 130 | | 256 x 256 | | 0.5 x 0.5 x 3.0 | 3 | 0.3 | 58 | 3000 | - | 199 | 04:23 |
| EPI diffusion b0_800 | | axial | 140 x 140 | | 90 x 90 | | 0.8 x 0.8 x 4.0 | 4 | 0.4 | 70 | 5550 | 2 | 1134 | 01:51 |
| After contrast injection : | |  |  | |  | |  |  |  |  |  |  |  |  |
| T1 SE fat saturation | | sagittal | 160 x 160 | | 256 x 192 | | 0.3 x 0.3 x 3.0 | 3 | 0.6 | 18 | 619 | - | 167 | 04:03 |
| T1 SE fat saturation | | axial | 170 x 170 | | 256 x 204.8 | | 0.3 x 0.3 x 3.0 | 3 | 0.6 | 16 | 679 | - | 190 | 03:01 |
| **Small FOV (small flex or purposes coils)** | | | | | |  | | | | | | | | |
| T2 STIR | | sagittal | 150 x 150 | | 256 x 256 | | 0.6 x 0.6 x 0.8 | 0.8 | - | 167 | 2000 | 2 | 444 | 07:06 |
| T1 TSE | | coronal | 100 x 100 | | 256 x 256 | | 0.2 x 0.2 x 2.5 | 2.5 | 0.25 | 14 | 1010 | 2 | 183 | 04:21 |
| T2 DIXON | | axial | 120 x 120 | | 320 x 288 | | 0.2 x 0.2 x 3.0 | 3 | 0.3 | 52 | 3150 | 2 | 319 | 04:28 |
| Diffusion Resolve b0_800 | | axial | 100 x 100 | | 160 x 128 | | 0.6 x 0.6 x 4 | 4 | 0.4 | 68/114 | 7110 | 2 | 558 | 04:10 |
| After contrast injection : | |  |  | |  | |  |  |  |  |  |  |  |  |
| T1 SE fat saturation | | coronal | 100 x 100 | | 256 x 256 | | 0.2 x 0.2 x 2.5 | 2.5 | 0.25 | 14 | 551 | 2 | 183 | 07:08 |
| T1 SE fat saturation | | sagittal | 100 x 100 | | 256 x 256 | | 0.2 x 0.2 x 2.5 | 2.5 | 0.25 | 14 | 551 | 2 | 183 | 03:36 |

Acquisition parameters depending on the anatomical location, FOV size, and coil chosen to fit the patient and the location of disease. In children younger than 6 years old, the initial sequence is an extended field of view three-dimensional STIR sequence.

*EPI* echo planar imaging, *FOV* field of view, *SE* spin echo, *STIR* short tau inversion recovery, *TE* echo time, *TR* repetition time, *TSE* turbo spin echo
